# Efficient human 3D localization and free space segmentation for human-aware mobile robots in warehouse facilities

**DOI:** 10.3389/frobt.2023.1283322

**Published:** 2023-10-11

**Authors:** Dimitrios Arapis, Milad Jami, Lazaros Nalpantidis

**Affiliations:** ^1^ Robotics Innovation Lab, Department of Robotics and Operational Technologies, Novo Nordisk A/S, Bagsvaerd, Denmark; ^2^ Group of Automation and Control, Department of Electrical and Photonics Engineering, DTU—Technical University of Denmark, Kongens Lyngby, Denmark

**Keywords:** human localization, free space segmentation, multi-task learning, mobile robots, warehouse robotics

## Abstract

Real-time prediction of human location combined with the capability to perceive obstacles is crucial for socially-aware navigation in robotics. Our work focuses on localizing humans in the world and predicting the free space around them by incorporating other static and dynamic obstacles. We propose a multi-task learning strategy to handle both tasks, achieving this goal with minimal computational demands. We use a dataset captured in a typical warehouse environment by mounting a perception module consisting of a Jetson Xavier AGX and an Intel L515 LiDAR camera on a MiR100 mobile robot. Our method, which is built upon prior works in the field of human detection and localization demonstrates improved results in difficult cases that are not tackled in other works, such as human instances at a close distance or at the limits of the field of view of the capturing sensor. We further extend this work by using a lightweight network structure and integrating a free space segmentation branch that can independently segment the floor space without any prior maps or 3D data, relying instead on the characteristics of the floor. In conclusion, our method presents a lightweight and efficient solution for predicting human 3D location and segmenting the floor space for low-energy consumption platforms, tested in an industrial environment.

## 1 Introduction

The advent of Industry 4.0 has brought about significant changes in the landscape of warehouses and production facilities, with automation and digitization playing a crucial role in streamlining operations and increasing efficiency. Mobile robots have emerged as indispensable components in these environments, assisting in tasks such as material handling, inventory management, and transportation. However, as the presence of mobile robots in industrial settings becomes more prevalent, so too does the potential for accidents and incidents involving humans, robots, and infrastructure. Ensuring the safety of human workers and minimizing these risks is a critical challenge that must be addressed. Modern warehouses present challenges due to intricate environments, highlighting the need for advanced perception and human-aware interfaces. Factors like narrow aisles, dynamic obstacles, and varying lighting complicate navigation and can jeopardize safety, especially with the coexistence of humans and machines. To this end, developing human-aware robots with advanced perception capabilities is of paramount importance. Our work seeks to address this critical challenge in industrial environments by developing a robust, efficient, and adaptable perception system. Retrofitting an advanced perception system can elevate the capabilities of existing mobile platforms in warehouses. Due to varied robot platforms, perception methods differ: some use LiDAR, others use cameras, and some AGVs rely on tactile sensors. These variations create disparities in accuracy and adaptability. Installing a supervisory perception module can standardize intelligence across all mobile agents, ensuring consistent navigation and detection quality. This not only addresses integration issues in diverse mobile robot fleets but also boosts their overall effectiveness, fostering a harmonized robotic workforce.

More precisely, in this work, we propose an efficient multi-task learning strategy for *human 3D localization* as well as for *free space segmentation*, specifically designed for robots operating in industrial environments such as warehouses and production facilities. Human 3D localization is a key prerequisite for socially-aware robots in industrial settings, as accurate localization enables them to perceive and predict human movements, thus facilitating safer and more effective collaboration between humans and robots. By employing pose estimation techniques, we localize humans in 3D space, enabling the robot to better understand the context of the interaction and adapt its behavior accordingly. Free space segmentation is essential for path planning and obstacle avoidance, particularly in cluttered industrial environments where potential hazards are abundant. By accurately identifying free space areas, our method allows robots to navigate shared spaces more effectively, preventing collisions and ensuring smooth and efficient operation in warehouses and production facilities.

Our approach combines these two tasks within a single lightweight model, making it suitable for implementation on low-energy consumption devices and enabling real-time operation on resource-constrained mobile robots. Utilizing the capabilities of multi-task learning, our unified network delivers results comparable to individual state-of-the-art networks for human localization and floor segmentation, all while maintaining reduced computational complexity. The proposed approach—based on multi-task learning—is adaptable and can be expanded to incorporate additional tasks, further enhancing the capabilities of socially-aware robots in the industrial domain.

The *contribution* of this work is threefold: 1) we propose a new lightweight model capable of combining 3D localization and free space segmentation from RGB images, while also incorporating any possibly available depth data to further improve its predictions, 2) we train our approach and test its performance in real industrial settings—warehouses, and 3) we examine the efficacy of multi-task learning approaches for achieving computationally cheaper implementations suitable for mobile robots. The code of this work is available at https://github.com/dimarapis/hamoro.

The remainder of the paper is structured as follows. We begin by discussing related work and providing an overview of the challenges and opportunities of enhanced perception capabilities in warehouse and production industrial mobile robots. Subsequently, we detail the methodology behind the two individual tasks and the multi-task learning strategy, present the lightweight model architecture, and discuss the results of our experiments. Finally, we explore the potential of our approach for enhancing the performance of mobile robots in industrial environments and outline future research directions.

## 2 Related work

### 2.1 Human modeling in computer vision and robotics

#### 2.1.1 Human pose estimation

Human pose estimation is an important task in computer vision and robotics. There are a number of methods, such as using depth cameras, skeleton-based models, and machine learning algorithms, that have made considerable strides in this area. Early works in human pose estimation focused on human part association with the most dominant techniques being based on pictorial structures (Andriluka et al., 2009; Felzenszwalb and Huttenlocher 2005) which however often failed to capture the correlation of deformable or occluded parts. In recent years, deep learning methods have emerged as a powerful tool for human pose estimation, revolutionizing the way researchers and technologists approach the challenges associated with understanding and interpreting human postures. Early work in this field, such as DeepPose from (Toshev and Szegedy, 2014) and stacked hourglass networks from (Newell et al., 2016), utilize Convolutional Neural Networks (CNN) to predict joint locations directly. However, these methods typically struggle with complex poses due to the limitations in capturing high-level spatial configurations. To tackle this, Graph Neural Networks (GNN) have been applied to model the dependencies among body joints. For instance, Yan et al. (2018) Spatial Temporal Graph Convolutional Networks (ST-GCN) proposed to represent human bodies as graphs, with nodes as body joints and edges as physical or temporal connections between joints. Despite these advances, these models were often trained and tested in controlled settings in single-person detection frameworks. To tackle the real-world variability and complexity and include multi-person pose estimation, the latest learning-based methods are often categorized into top-down methods and bottom-up methods. Top-down methods, such as HRNet (Sun et al., 2019) and AlphaPose (Fang et al., 2022), first obtain a set of bounding boxes of people and then predict human poses. On the other hand, bottom-up methods, such as OpenPose (Cao et al., 2018) and OpenPifPaf (Kreiss et al., 2021), predict all 2D joints and then assemble them into independent skeletons. Typically, top-down approaches yield higher accuracy for distinct individual detections, yet underperform when faced with overlapping or closely grouped individuals. Their computational time is roughly tied to the number of people in the image, potentially resulting in substantial processing time. In our study, we use the YOLOv8-pose network, a top-down pose estimation model built upon YOLO-pose (Maji et al., 2022). Given that our context does not encompass large crowd scenarios or overlapping detections, the usage of bounding box detections proves advantageous. This selection has yielded impressive results in real-world evaluations, as evidenced by the detection examples illustrated in Section 4.1.

#### 2.1.2 Human 3D localization

Human 3D localization, the task of determining a person’s spatial location in three dimensions, is critical for tasks such as autonomous robot navigation and human-robot interaction. There are various techniques used such as LiDAR-based (Yin et al., 2021), monocular (Ku et al., 2019; An et al., 2021) and stereo-based 3D localization (Bertoni et al., 2020). Recent works utilize deep learning methods on single image frames and can produce 3D bounding boxes (Bouhsain et al., 2020), 3D pose estimation (Pavlakos et al., 2017), and 3D localization given known 2D poses (Bertoni et al., 2019). Building on a foundation similar to ours, Rolley-Parnell et al. (2018) investigated the use of an affordable RGB-D sensor to transform 2D skeleton pose obtained using OpenPose into a 3D pose for robot teleoperation. Although their study diverged in domain and did not utilize depth points within the deep learning approach, it shared our challenge of filtering out invalid depth values and showcased a scenario where RGB and depth can be effectively combined for human 3D localization. Each of the aforementioned methods has distinct drawbacks, such as cost inefficacy, low accuracy when occlusions exist, or difficulties in acquiring sufficient high-quality data for training (Iftikhar et al., 2022). The present study aims to tackle these challenges by integrating 2D detection—benefiting from training data from past research and improved occlusion handling—with 3D data to address edge cases. Our framework uses 2D poses and any auxiliary depth data, if available, to fine-tune the predicted human 3D localization. By recognizing the strengths and limitations of the previous works, our goal is to offer a simple and robust solution within the realms of computer vision and robotics in industrial environments.

### 2.2 Free space segmentation

In the context of path planning and obstacle avoidance for mobile robots, most commercially matured frameworks have been based on LiDAR technology (Hebert, 2000; Huang et al., 2023). The primary constraint of 2D LiDAR sensors is their field of view at a fixed height, which overlooks one dimension and frequently leads to a failure in identifying obstacles, e.g., forklift tines (Chen et al., 2020). On the other hand, although 3D LiDAR is a reliable solution in identifying obstacles, and has been widely adopted by the research community, the high cost of such sensors has limited their wide application in mobile robotics (Yang et al., 2022). Additionally, both 2D and 3D LiDAR technologies do not contain as dense information as RGB sensors making it harder to categorize obstacles into specific objects, in cases where that is a necessity (e.g., differentiating between static and dynamic objects) (Yasuda et al., 2020). Fueled by the advances in deep learning, considerable effort has been put towards developing algorithms utilizing RGB and RGB-D data for free space identification and, subsequently, path planning and obstacle avoidance (Li and Birchfield, 2010; Zhao et al., 2018; Seichter et al., 2021). Deep learning-based methods can be used stand-alone to reduce costs compared to a LiDAR sensor suite (Messiou et al., 2022; Dang and Bui, 2023), or as a supervising method to improve the perception capabilities of mobile robots (Juel et al., 2018; Liu et al., 2021; Arapis et al., 2023). Despite these advancements, certain challenges persist, including segmenting free space under varying floor conditions and further reducing computational requirements for real-time applications. Previous works have also highlighted the manual labor involved in creating pixel-wise annotations as the bottleneck of training networks for free space segmentation (Tsutsui et al., 2018).

### 2.3 Multi-task learning in robotics

In the domain of robotics, the ability to perform multiple tasks concurrently is an area of substantial interest (Vandenhende et al., 2021; Zhang and Yang 2022). Multi-task learning (MTL) approaches aim to improve the performance of multiple related tasks by leveraging the shared information among them and at the same time, often reduce the computational requirements, by sharing some computations across tasks (Caruana, 1997). The MTL paradigm has been used extensively in various applications in robotics, including manipulation and perception (Zhang et al., 2019; He and Ciocarlie, 2021). Despite the significant advancements in the field, challenges persist. Particularly, identifying task relationships and selecting the appropriate level of task sharing are non-trivial and require more sophisticated methodologies (Zamir et al., 2018; Fifty et al., 2021). Each configuration may yield disparate outcomes, underscoring the necessity to rigorously evaluate typical multi-task balancing techniques within each specific setup, encompassing the tasks involved, dataset characteristics, and model complexity (Standley et al., 2019; Zamir et al., 2020). As highlighted by Seichter et al. (2020) in their work on multi-task learning for person detection, posture classification, and orientation estimation, balancing the tasks becomes harder the more heterogeneous the tasks are, e.g., mixing both regression and classification tasks. Various works underscore the importance of physical testing, as relying solely on previous works may overlook unique aspects of the current configuration, thereby potentially leading to inaccurate or sub-optimal results (Kendall et al., 2017; Liu et al., 2019). In our work, we will test various task-balancing techniques aiming for the best overall predicting performance for both heterogeneous tasks, with the overall goal of minimizing the computational requirements by sharing part of the network. An overview of these techniques along with references can be found in the work of (Senushkin et al., 2023).

### 2.4 Socially-aware robots in industrial environments

The deployment of socially-aware robots in industrial environments is a burgeoning field, with an emphasis on creating robotic systems that not only perform tasks efficiently but also seamlessly integrate into human-inhabited environments. To ensure the seamless integration of socially-aware execution in mobile robot tasks, the process entails a careful orchestration of multiple elements. This includes the sensor suite selection (Bonci et al., 2021), perception techniques (Charalampous et al., 2017), navigation efficiency (Gao and Huang, 2022), and comprehensive modeling of human behavior and ethical considerations (Tapus et al., 2019). Beyond the realms of perception and planning, some researchers have turned their attention to the pivotal task of communicating robot actions to surrounding humans. It is crucial to have systems like dialogue and projection as they provide intuitive and effective means of communication, bridging the gap between humans and robots, and ensuring seamless and safe interactions in dynamic environments like warehouses (Lopez Alaguero et al., 2022). Despite these advances, the application of socially-aware robots in industrial environments is hindered by significant challenges. These include the need for real-time perception and decision-making, the need for collecting high-cost domain-specific datasets, and the absence of specific metrics to evaluate their performance (Mavrogiannis et al., 2021). In this work, we will focus on the perception part aiming to identify the presence and 3D location of humans in the industrial environment. Human localization enhanced with free space segmentation will allow mobile agents to function effectively and safely in industrial environments.

## 3 Methodology

### 3.1 Overview of tasks

#### 3.1.1 Human 3D localization

**FIGURE 1:**
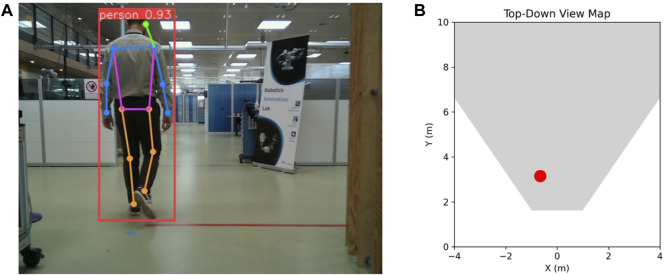
Example of **(A)** human detection with bounding box and pose estimation with 2D keypoints and **(B)** human localization on a top-down view, with the red dot representing their ground truth location. Please note that unless specified otherwise, this and the following illustrated test frames are not derived from real warehouse settings because of company privacy concerns related to those environments. Instead, we only illustrate test frames derived from a robotics testing facility.

Human 3D localization entails accurately determining a person’s position within a three-dimensional space. We anchor our approach on a 2D human keypoint estimation methodology. These keypoints, once identified, are processed using our algorithm which translates them into an estimation of the distance between the human subject and the sensor. The decision to primarily utilize RGB was influenced by considerations of scalability, sensor heterogeneity across varied robotic agents, and the limitations of cost-effective depth sensors, such as missing points and limited sensing range. Nevertheless, for the final step of human localization, available depth data is integrated to enhance accuracy. The details of our network are elaborated in Section 3.4. Figure 1 showcases a frame capturing the detected human alongside a top-down view map illustrating the human’s ground truth location.

#### 3.1.2 Free space segmentation

**FIGURE 2:**
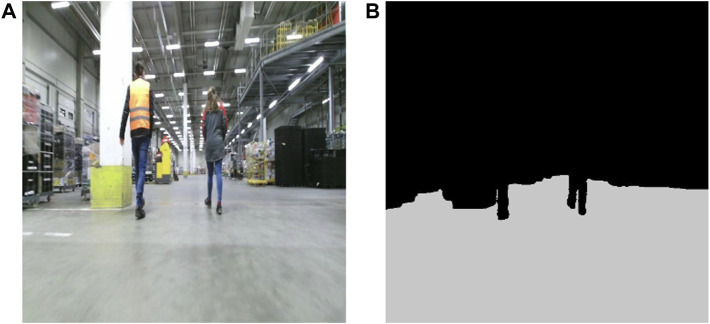
Sample of **(A)** RGB image and **(B)** corresponding free space segmentation on the image plane. The RGB sample belongs to an online warehouse image repository that can be found through the provided link in Section 3.1.2.

Free space segmentation plays a crucial role in path planning and obstacle avoidance, enabling autonomous robots to navigate safely in complex environments. Given a monocular image as input (Figure 2A), it involves the task of classifying each pixel in a scene as traversable space or obstacle (Figure 2B)—image sample taken from (https://universe.roboflow.com/divya-tiwari-u2mrc/warehouse-vemit). For the task of free space segmentation, we use a monocular image and modify an existing segmentation network to output two classes, resulting in a binary segmentation task. It is essential to highlight that our choice of RGB images over low-cost RGB-D sensors is strategic. We observed that low-cost RGB-D sensors frequently struggle to capture depth in reflective zones, common in environments like factories and warehouses. Given these challenges, we decided that segmenting on pure RGB images would yield more reliable results for our application.

### 3.2 Data

**FIGURE 3:**
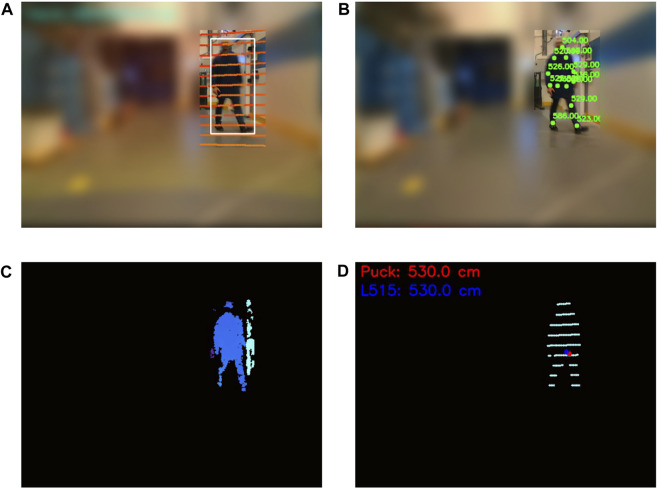
Example of data collection for 3D human localization. This frame has been derived from an actual warehouse environment. Parts of the images have been intentionally blurred due to data restrictions. **(A)** RGB image and overlaid 3D LiDAR scans, **(B)** RGB image with partial overlaid 2D keypoints and corresponding depth camera values, **(C)** depth map of the area within the bounding box, and **(D)** foreground 3D LiDAR points used for groundtruth distance.

Due to the nature of the problem, which is to solve two different tasks, there is a need to create a dataset that can cover both human localization and free floor segmentation. To accelerate the annotation process we chose two methods for automatic annotation, one for each task, and then filtered out any wrong annotated data. To collect the human localization dataset, we equipped a mobile robot (MIR100) with a perception module consisting of an edge computing unit (Jetson Xavier AGX), a depth camera (Intel L515), and a 3D LiDAR (Velodyne Puck) and deployed it in two Novo Nordisk environments, a warehouse and a robotics testing facility. We used the depth camera to collect RGB-D images, which we divided into 1) RGB (Figure 3A) used for 2D human keypoint estimation and free floor segmentation, and 2) depth map used to extract the auxiliary depth points of the human (Figure 3C) only to be injected in the network when fine-tuning the predicted human 3D localization. We observed absence and inaccuracies of depth for certain detected keypoints which was influenced by the precision of the 2D keypoint estimator and the distance between the human and the sensor. In order to filter out these values, we implemented a validation process in which the surrounding pixels within a 5 × 5 pixel window are considered. The condition for validation is that these neighboring pixels are in agreement, collectively forming a consistent pattern. This approach ensures that only coherent and compatible values are retained while discarding any outliers or inconsistencies (Figure 3B). It is important to highlight that using a fixed window of pixels presents challenges especially given the variable distance of the camera from surfaces. Ideally, considering nearest neighbors in the point cloud space would offer a more refined solution. However, given our study’s data scope and volume, the chosen method met our needs effectively. We used the 3D LiDAR data points to compute the groundtruth distance of the human (Figure 3D). We initiated the process by filtering out points outside the detected bounding box and then clustering the remaining points into the foreground and background. The final groundtruth value is the median value of the foreground points. The drawback of this approach is that it does not take into account the deformations of the human body, which can lead to measurement errors. While comparing with the average value of the 3D keypoints derived from the camera LiDAR sensor, an observed discrepancy of up to 20 cm came to light. Nonetheless, we acknowledge a slight possible variation between our computed groundtruth distance and the person’s actual distance due to the automated annotation process. Additionally, considering our practice of extending the predicted 3D location to create a safety margin around the detected person, any such differences are likely to be absorbed or rendered insignificant.

**FIGURE 4:**
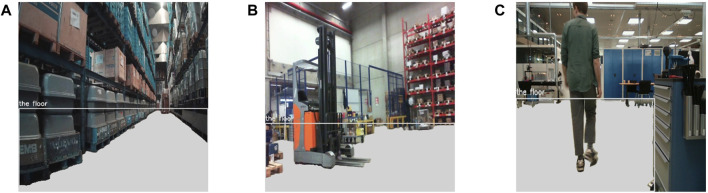
Samples images with overlaid floor segmentation (in gray color) based on GroundingDino prompt and SAM. Samples **(A)** and **(B)** belong to the online warehouse dataset and sample **(C)** is captured in our testing lab.

For the training dataset of the free floor segmentation task we first downloaded the raw images of an online warehouse dataset which has been used for object detection (https://universe.roboflow.com/divya-tiwari-u2mrc/warehouse-vemit) and then automatically generated annotations using Grounding DINO (Liu et al., 2023) and Segment Anything Model (SAM) by Kirillov et al. (2023). GroundingDino is an open-set object detector, that can detect any object in the scene, given a text prompt. The output of Grounding DINO is a 2D bounding box enclosing the object of interest. SAM is a segmentation model that can create segmentation masks in an image, without any input. In our case, we used the bounding box detection as an input for SAM and used the mask with the highest probability in the bounding box as seen in Figure 4. For our testing dataset, we use the mobile robot setup described during the human dataset collection process and use these images for free floor segmentation data, creating the groundtruth segmentation mask using the same automatic annotation process as the training dataset of free floor segmentation. For all images in the dataset (including these samples) the prompt used was “the floor.”

**FIGURE 5:**
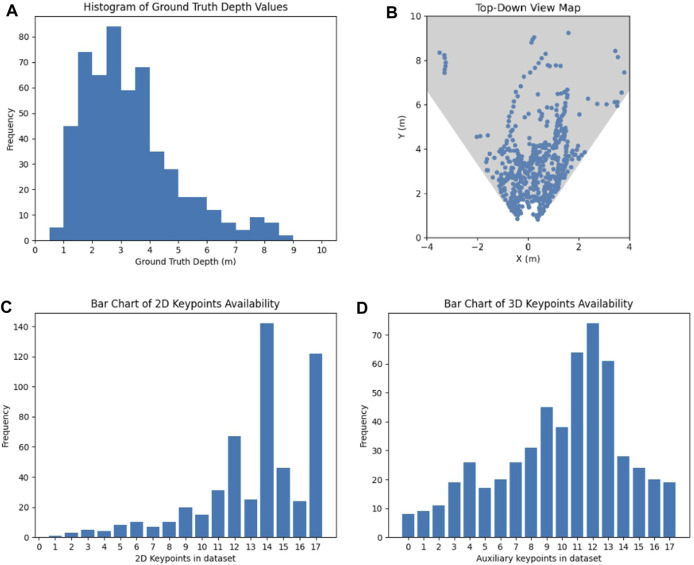
Analysis of the gathered dataset: **(A)** Histogram of observed groundtruth depth values, **(B)** positions of humans in the dataset images visualized on a top-down view of the robots field of view, **(C)** bar chart of keypoints with a confidence higher than 20%, and **(D)** bar chart of keypoints with additional depth values.

We decided to use the robotics testing facility environment as testing dataset to gauge the algorithms’ generalization potential and visualize our results of both tasks, given that this environment is unclassified. Information about the dataset including the distribution of human location in the world, ground truth distance of human detections and 2D and 3D keypoint availability is summarized in Figure 5.

### 3.3 Metrics

#### 3.3.1 Human 3D localization metrics

To evaluate human 3D localization we use the mean absolute error (mAE) of prediction—see Eq. 1. For each frame *i*, we compute the absolute error between its predicted distance *d*_*pred*_[*i*] and the groundtruth distance *d*_*gt*_[*i*] of the human instance from the sensor and average the measurements for a final mAE metric. In our human localization framework the groundtruth and predicted data points are collinear, traced along a vector stemming from our depth camera sensor to the furthest data reference. Given this linear alignment, the mean absolute error (mAE) and the Euclidean distance, which is another commonly used metric, effectively yield identical values.

$$\text{mAE}=\frac{1}{n}\overset{n}{\underset{i=1}{\sum }}\left|d_{\mathrm{pred}}\left[i\right]-d_{gt}\left[i\right]\right|$$ (1)

The lower the mAE, the closer the predicted values are to the actual values, and hence the better the predictive model. To account for the complexity of the task which relies on the 2D keypoints availability and accuracy of the 2D keypoints detector, we also evaluate the individual absolute error of each frame based on the 2D keypoint availability and the distance from the sensor, the results of which can be seen in Section 4.1.

While the mAE metric effectively summarizes our network’s performance, we also calculate the maximum absolute error (maxAE)—see Eq. 2. This metric highlights the utmost error that could arise in prediction values. Within our specific case and context, when comparing two models, we would prioritize a model with a significantly smaller maximum error over one with only a slightly lower mAE, as the latter can be corrected with a slightly larger safety layer around the prediction.

$$\text{maxAE}=\overset{n}{\underset{i=1}{\max}}\left|d_{\mathrm{pred}}\left[i\right]-d_{gt}\left[i\right]\right|$$ (2)

#### 3.3.2 Free space segmentation metrics

To evaluate free space segmentation we use the Jaccard Index, also known as the Intersection over Union (IoU), which is a widely adopted metric for semantic segmentation tasks—see Eq. 3. It is used to quantify how well the predicted segmentation aligns with the grounth-truth label.

The Intersection over Union (IoU) for binary segmentation is defined as the ratio of the intersection between the predicted pixels (A) and the ground truth pixels (B) to their union, given by:

$$\text{IoU}=\frac{A\cap B}{A\cup B}$$ (3)

where *A* is the set of predicted pixels, and *B* is the set of ground-truth pixels for the class under consideration.

The mIoU metric accounts for both false positives and false negatives, providing a comprehensive view of the segmentation algorithm’s performance across the two different classes, free space and occupied space. A value of 1 indicates a perfect match between prediction and ground truth, whereas a value of 0 represents no overlap.

To underscore the significance of obstacle avoidance, we further explore the recall of the model on the obstacle class (ObstacleRec)—see Eq. 4. Recall holds significant importance as it sheds light on the model’s effectiveness in correctly identifying obstacle pixels while minimizing the likelihood of missing any obstacles. Recall, also known as the True Positive Rate, quantifies the proportion of true positive predictions (correctly identified obstacle pixels) in relation to the total number of actual positive instances (obstacle pixels) present in the ground truth.

$$\text{ObstacleRec}=\frac{\text{Obstacle True Positives}}{\text{Obstacle True Positives }+\text{ Obstacle False Negatives}}$$ (4)

A high recall score signifies that the model is adept at capturing a substantial portion of the obstacle regions, emphasizing its ability to avoid missing obstacles, which is crucial for ensuring safe and comprehensive obstacle detection in robotics.

#### 3.3.3 Multi-task learning metrics

Due to the presence of multiple tasks and corresponding metrics, we employ the Δ_MTL_ metric introduced in the study by Liu et al. (2022). This metric combines the metrics from each task to comprehensively assess the overall performance of the multi-task learning model. Δ_MTL_ is a flexible metric and one can choose more than one metric per task to assess the performance of the multi-task learning setup. In our case, we evaluate the difference between the human 3D localization mAE metric and the mean Intersection over Union (mIoU) for free space segmentation, compared to the values achieved by the individually trained networks, as seen in Eq. 5.

$$\Delta_{\text{MTL}}=\frac{{\text{mAE}}_{\text{single}}-{\text{mAE}}_{\text{MTL}}}{{\text{mAE}}_{\text{single}}}-\frac{{\text{mIoU}}_{\text{single}}-{\text{mIoU}}_{\text{MTL}}}{{\text{mIoU}}_{\text{single}}}$$ (5)

The resulting Δ_MTL_ produces a single metric representing the percentage of improvement. Negative values of Δ_MTL_ indicate a network that performs worse than the individually trained networks.

### 3.4 Architecture

**FIGURE 6:**
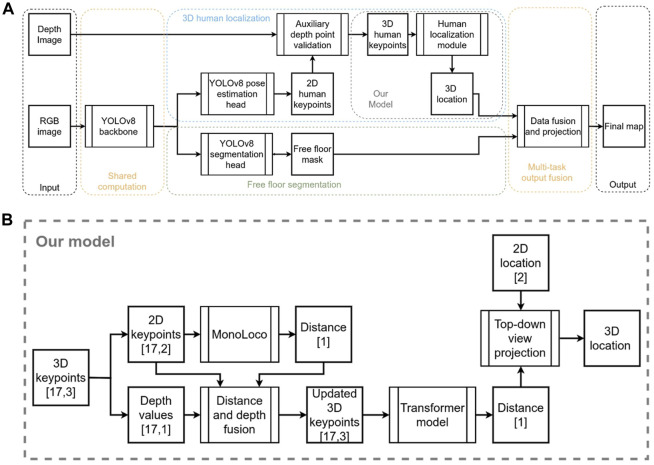
**(A)** Block diagram of network architecture. **(B)** More detailed block diagram of the proposed human localization module.

In this section, the network architecture will be presented, complemented by an illustration of the different modules used (see Figure 6A). An RGB image is fed into the YOLOv8 shared backbone, which contains the first 22 layers, up to and including the last C2f layer of the network. Then, we have added two individual heads, each responsible for one task. For the pose estimation head, we use the last layer of YOLOv8-pose which outputs the 2D human keypoints. We use the predicted keypoints and combine them with the depth image into an auxiliary depth point validation module to filter out inaccurate depth points and get 3D human keypoints. The 3D human keypoints are 17 keypoints based on COCO keypoints format with x, and y positions in the image and a depth value—the tensor shape is [17,3]. In many cases, keypoints do not have a corresponding value in the depth image, due to missing depth values in that region or in case it was filtered out as noisy depth data, and in that case the keypoint is associated with a depth value of 0. The subsequent module introduces our novel human localization algorithm, often referred to as “our model” in preceding and subsequent sections. Our model draws inspiration from Monoloco but incorporates significant modifications to harness auxiliary depth measurements. The novelty of these modifications aims to bolster predictions in scenarios where Monoloco was less proficient, particularly when the human subject was heavily obscured or off-frame. Figure 6B illustrates our innovative approach to 3D human localization. We partition the 3D keypoints into a tensor of 2D keypoints and another tensor holding their associated depth values. The 2D keypoints are channeled into the Monoloco component, yielding a distance prediction. This prediction assists in substituting absent depth values within the depth tensor. Following that, it merges with the 2D keypoints tensor to produce an enriched 3D keypoints tensor. Subsequently, a transformer model is engaged to refine the 3D keypoints tensor and provide an updated distance metric which, when combined with the human’s 2D image plane coordinates, is projected into a top-down view, concluding in a precise human location.

For the task of segmenting free space in our application, we make use of imagery from a monocular camera setup. Recognizing the need for accuracy in this task, we have chosen to adapt the YOLOv8-seg segmentation network, which is known for its performance. Given that our primary objective is to distinguish free space from other areas, we modified the network to cater specifically to a binary segmentation scenario. Hence, instead of the usual multi-class outputs, the adapted YOLOv8-seg now produces results for just two classes, streamlining the task to better suit our requirements.

The lightweight nature of our design is a direct consequence of two crucial decisions. Across the range of state-of-the-art methods, YOLOv8 emerged as a front-runner due to its computational efficiency combined with robust accuracy. It outpaces competitors like OpenPifPaf, which was used in the original Monoloco design, in terms of speed, ensuring swift operations even on resource-limited devices. Secondly, our choice of a multi-task approach leads to fewer network parameters and faster inference, ass seen in Section 4.3. Significantly, the shared architecture across the majority of the network plays a crucial role in achieving this lightweight configuration. Collectively, these choices attest to our dedication to crafting a design that epitomizes both efficiency and efficacy.

### 3.5 Training and testing framework

We train the entire model on a single NVIDIA RTX 3090 32 GB GPU with a batch size of 16 and initial learning rate of 10^–4^ using the Adam optimizer. We train for 300 epochs and use a step scheduler to decrease the learning rate to half every 100 steps. Because of the distinct tasks and data collection approaches, we employ separate datasets to train the network for each task. To mitigate the challenges stemming from the varying sizes of these two datasets, we curate a subset of the initial free space segmentation dataset. This subset is tailored to match the dimensions of the human localization dataset, and we ensure uniform batch sizes across both datasets for training. During the multi-task learning model training, we alternate between the two datasets iteratively and combine the losses of both tasks prior to executing the backward propagation step. As our testing platform, we use the NVIDIA Jetson Xavier AGX.

## 4 Results

### 4.1 Human localization

In this section, we present the evaluation results of our model and a comparison with Monoloco. The comparative quantitative analysis on our collected dataset demonstrates a significant improvement in human localization for the majority of the data samples, as seen in Table 1. When testing across the total testing dataset, we reduced the mAE to more than half compared to Monoloco, and also improved on the maxAE.

**TABLE 1:** Human localization results for various distance ranges in our collected dataset.

	Range					
	All distances		0–3 m		3 m-max	
Method	mAE *↓*	maxAE *↓*	mAE *↓*	maxAE *↓*	mAE *↓*	maxAE *↓*
Monoloco	0.455	2.19	0.616	2.19	0.241	0.68
Our model	0.185	1.81	0.228	1.81	0.128	0.52

**FIGURE 7:**
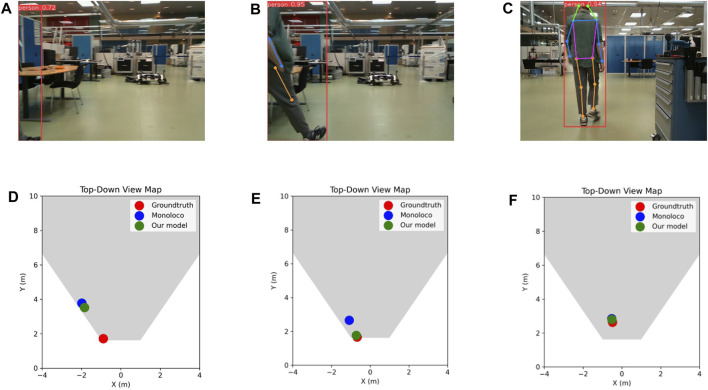
**(A–C)** Example frames from the testing dataset and **(D–F)** corresponding top-down views showing the groundtruth human position, as well as Monoloco’s and our model’s predictions. Frame **(A)** constitutes the most problematic case in the whole testing dataset where both examined approaches completely fail to localize the barely detected human, whereas **(B)** and **(C)** constitute two typical cases.

Upon closer inspection of the dataset, and more specifically, the data point that produced the maximum absolute error (maxAE) for both models, we observed a significantly challenging scenario that is visualized in Figure 7A. It can be seen that the 2D human keypoint algorithm detected a person when only a fraction of their body is visible in the frame, and subsequently, only one 2D keypoint. This is a very challenging scenario for the localization algorithm, which can be seen in the resulting estimation of Figure 7D.

On the next frame (Figure 7B), our model is able to improve upon Monoloco on close detections and when the 2D keypoints availability is low (Figure 7E). Finally, in Figures 7C,F, it is apparent that both models localized the human with very high accuracy, given a full view of the skeleton and high 2D keypoint availability for Monoloco and additional 3D keypoint availability for our proposed model.

**FIGURE 8:**
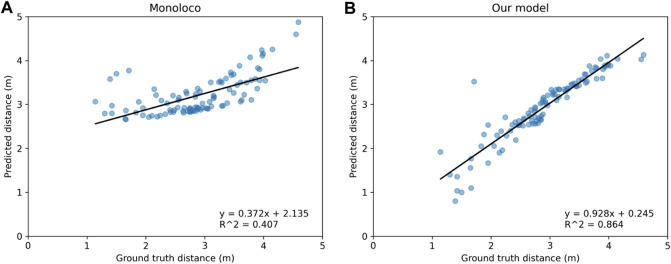
Comparison of predicted distances for detected humans against their groundtruth distances for **(A)** Monoloco and **(B)** our method. As the linear fitted models reveal, our approach gives distance estimations much closer to their groundtruth values.

When plotting the predicted values against the groundtruth ones, one should ideally expect a linear fit of the data points that have a slope of 1 and an offset of 0, with the R-squared value being 1. In the comparative analysis of the two models visualized in Figure 8, the slope and offset values are valued at 0.928 and 0.245 respectively for our model compared to 0.372 and 2.135 for Monoloco. The calculated R-squared value of 0.864 for our model signifies a notably stronger linear relationship between the predicted distances and the ground truth distances. This indicates our model’s ability to more closely predict the true distances. In contrast, the R-squared value of 0.407 for the Monoloco model suggests a weaker linear fit, indicating a less precise correspondence between its predictions and the actual distances. The substantial difference in R-squared values underscores the superior predictive performance of our model which, combined with the slope and offset values, reinforces its potential for accurate and consistent distance estimation.

**FIGURE 9:**
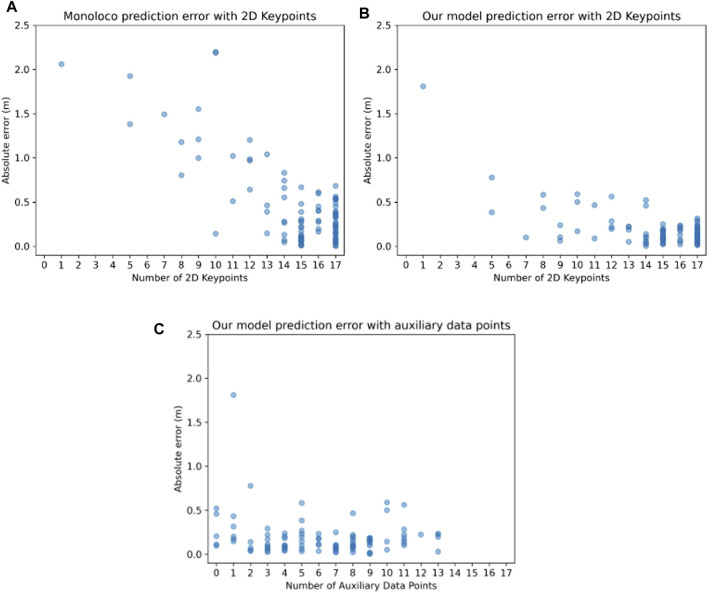
Relationship between the localization absolute error and the number of 2D points in an image for **(A)** the Monoloco and **(B)** our method. One can observe that our approach results in localization predictions whose absolute error is relatively independent of the number of available 2D points. In **(C)**, the relationship between the localization absolute error and the number of auxiliary data points for our method is depicted—Monoloco cannot incorporate information from auxiliary data points, therefore only our own approach is shown here. One can observe that even if no auxiliary data points are available, our approach can still provide localization predictions of low absolute error.

Upon observing certain patterns in the predictions for both models, particularly the pronounced errors when 2D keypoints availability was lower and in scenarios where the person was in close proximity, we opted to conduct a more extensive investigation. In Figure 9 we compare the performance of the two models with respect to the amount of available 2D keypoints they receive. It should be noted that the 2D keypoints network predictor (YOLOv8-pose) will always output 17 keypoints that represent the whole body, by assigning a low confidence value and x,y pixel coordinates at the image edges for keypoints that are outside of the image boundaries. Therefore, when evaluating the model, we only use keypoints with a confidence higher than 20%. As a general rule, if there are only a few keypoints available, the human might be very close to the sensor, on the edge of the field of view, or both. Naturally, the fewer the keypoints, the harder it is for the network to make a correct prediction, which is also visualized in Figure 9A. As seen in Figure 9B, our model is able to make better predictions by incorporating the auxiliary depth points.

To assess the degree to which auxiliary depth points contribute to improved predictions for our model, we generate the plot depicted in Figure 9C. This plot illustrates the relationship between the absolute prediction error and the quantity of accessible auxiliary points.

The results show that the prediction error exhibits a consistent trend across a spectrum of different quantities of supplementary data points, thus showcasing the model’s ability to benefit from the auxiliary points, regardless of the amount of auxiliary data integration. Interestingly the model learns to make correct predictions even in the case of 0 extra data points, showing its generalization capabilities.

### 4.2 Free space segmentation

In this section, we present the results of the free space segmentation network. For our testing dataset, the YOLOv8-seg model yielded a mean Intersection over Union (mIoU) of 0.859 and an obstacle recall (ObstacleRec) of 0.898, as seen in Table 2. In addition to the mIoU, it is equally critical to understand the extremities of performance for robust evaluation. To offer insights into the potential edge cases, we report both the minimum and maximum IoU values from our testing dataset. The sample with the lowest segmentation performance yielded an IoU of 0.764, whereas the best-performing sample achieved an IoU of 0.932. Highlighting these values serves to provide a holistic perspective of the model’s consistency, range, and potential limitations.

**TABLE 2:** Free space segmentation results.

Method	mIoU *↑*	ObstacleRec *↑*
YOLOv8-Seg	0.859	0.898

**FIGURE 10:**
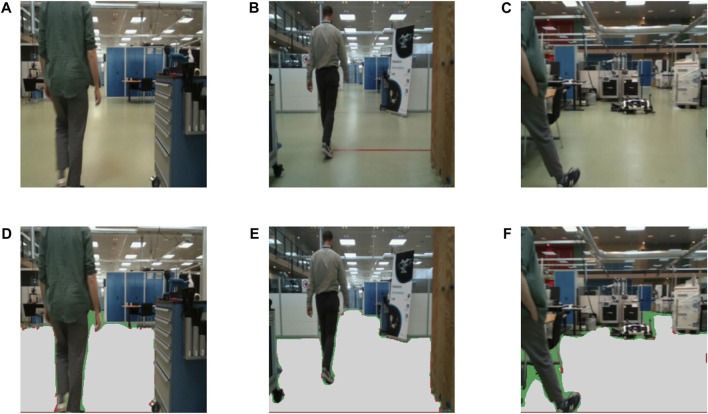
Visual free space segmentation results. **(A–C)** Example frames from the testing dataset and **(D–F)** corresponding free space segmentation predictions. The gray-colored pixels represent the true positives, green colored pixels show the false negatives and the red pixels show false positives for the free space class.

An illustration of the segmentation results on three samples can be seen in Figure 10. In the subfigures d,e, and f the predicted mask is overlaid with three colored masks, gray, green, and red, for the true positives, false negatives, and false positives respectively. It is self-evident, that in case of a misclassification, false negatives are preferable, as a false positive (classifying an obstacle as a free space) can lead to actual collisions. Upon visual inspection, the model effectively segments free space, particularly the areas crucial for navigation. However, it tends to be cautious in tighter spaces, which would be impractical to navigate anyway. This results in instances, represented by the green values, where the model mistakenly classifies the space as an obstacle when the groundtruth data indicates it as open space. Notably, only a handful of pixels near the edges of actual objects are incorrectly marked as open areas although they are, in fact, obstacles. The obtained results demonstrate the efficacy of the model in the free space segmentation task.

### 4.3 Multi-task learning

We evaluate the efficacy of widely used techniques within the realms of computer vision and robotics within our particular context. While the tasks we have selected for integration through multi-task learning hold the potential to mutually enhance planning, navigation, and other robotic strategies, these tasks inherently lack substantial shared information and mutual benefits. This makes achieving a harmonious balance between the two tasks a notably challenging endeavor.

In Table 3, the comparative analysis of multiple MTL balancing techniques is presented. As seen in the last column of the table (Δ_*MTL*_), none of the tested strategies can balance out the losses in a way that would improve the total performance of both tasks. Upon closer inspection, it can be seen that there is no single metric that has improved by deploying the MTL strategy. The findings indicate that in this scenario one task can not transmit information to the other task in a way that would improve that task’s performance. The multi-task balancing techniques show an inferior performance of up to 15.1%, with the best performing technique being uncertainty weighting with a minor 3.1% drop in performance.

**TABLE 3:** Comparison of task balancing methods.

	Human localization		Free space segmentation		Total
	mAE *↓*	maxAE *↓*	mIoU *↑*	ObstacleRec *↑*	Δ_*MTL*_ *↑*
Task-weighting balancing methods					
Equal Weights (EW)	0.192	1.831	0.836	0.846	−6.5
Dynamic Weight Averaging (DWA)	0.193	2.078	0.833	0.808	−7.4
Geometric Loss Strategy (GLS)	0.193	1.928	0.843	0.811	−6.2
Random Loss Weighting (RLW)	0.199	2.105	0.794	0.803	−15.1
Uncertainty Weighting (UW)	0.187	2.102	0.842	0.845	−3.1
Gradient-based balancing methods					
Gradient Normalization (GradNorm)	0.191	1.976	0.757	0.867	−15.1
Conflict-averse Gradients (CAGrad)	0.189	2.113	0.846	0.871	−3.7
Gradient sign Dropout (GradDrop)	0.197	1.930	0.826	0.806	−10.3
Gradient Vaccine (GradVac)	0.199	2.171	0.799	0.796	−14.6
Gradient Surgery (PCGrad)	0.197	1.925	0.826	0.819	−10.3

Our primary focus lies in the processing aspect of these tasks, aiming to harness the potential of multi-task learning to enhance the overall efficiency and effectiveness of the computational procedures involved. To this end, we experience a significant decrease in computational requirements in both terms of network parameters but also in processing time. In Table 4, we present the parameters (in millions, M) and inference time (in milliseconds, ms) for both single-task modules and our multi-task network across two hardware setups. The efficiency of our multi-task approach is evident, requiring only 55.7 M parameters and a rapid inference time of 31 ms. In contrast, handling the two tasks separately demands a total of 91.95 M parameters and 48 ms inference time on the NVIDIA RTX 3090 GPU—showcasing a reduction of 39% in parameters and 35% in time. The lightweight advantage of multi-task learning becomes even more pronounced on the power-efficient NVIDIA Xavier AGX device. Using the more extensive YOLOv8 model variant, processing the tasks separately took 325 ms per image. But with the combined tasks in our multi-task configuration, we saw a remarkable drop to 202 ms—a reduction of 37.8% in processing time. It should be noted that for smaller variations of YOLOv8, the inference time would be lower, however, the performance would also be affected.

**TABLE 4:** Comparison of inference time and model size. We report the metrics of the large (L) variation of YOLOv8, as this gave us a good balance between performance and computation, and it is the dominant variation used across our experiments.

			3090 GPU	Xavier AGX
Task	Model	params (M) *↓*	Inference (ms) *↓*	Inference (ms) *↓*
Single-task network				
2D keypoint estimation	YOLOv8-Pose	44.48	20	142
3D Human localization	Our Model	1.47	3	16
Free space segmentation	YOLOv8-Seg	46	25	167
Sum of single tasks	-	91.95	48	325
Multi-task network				
Joint prediction	Full architecture	55.7	31	202

### 4.4 Fusion

**FIGURE 11:**
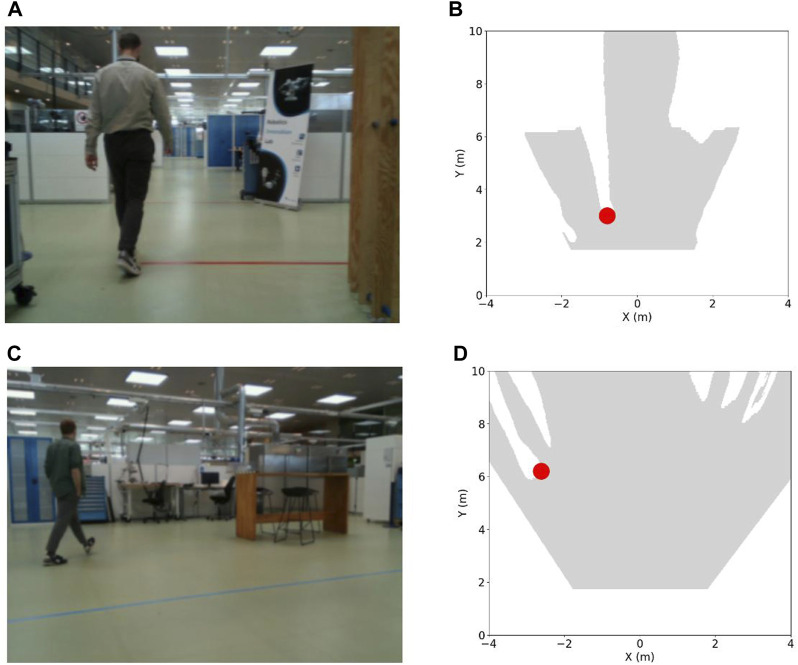
**(A,C)** Image samples and **(B,D)** top-down perspectives of the environment corresponding to the image samples. The open space is depicted as a gray-colored area, while detected human locations are marked with red-colored dot.

We move forward by integrating the results of the two tasks to produce a comprehensive top-down perspective of the environment, showcasing both the open space and the pinpointed locations of detected humans. Importantly, this data integration is not contingent upon the multi-task framework and remains versatile. Thus, it can be applied irrespective of whether we adopt a multi-task learning approach or handle tasks separately. Figure 11 presents a clear top-down representation of the environment, highlighting the available free space contrasted with the precise human locations. This visual representation offers an intuitive grasp of the spatial dynamics within the facility. The distinction between open areas and human presence is made visually evident, allowing for immediate recognition and space utilization. The delineated human positions, marked with a distinct color, stand out against the backdrop of free space and serve as a powerful tool for understanding human-robot interaction within the facility, paving the way for informed decision-making related to safety enhancement and space management.

## 5 Conclusion

### 5.1 Summary of key findings and contributions

We presented a 3D human localization model that builds upon Monoloco and can use auxiliary depth points, if available, to improve its prediction. The model performs exceptionally well on out-of-distribution data—cases including close detections, detections at the edge of the field of view of the capturing sensor, and detections with very few available 2D pose keypoints. We additionally trained a segmentation network to identify free space, using state-of-the-art foundation models in an automated annotation process and showed the feasibility of using such models to accelerate the creation of warehouse datasets.

In our multi-task computer vision scenario, using multi-task learning did not lead to a performance boost, but rather resulted in a slight performance decrease. However, it significantly improved computation time. This reduced computational burden offers an opportunity to incorporate additional tasks or extend the network in new directions. Depending on the computational capacity and the required accuracy of predictions, one should weigh the advantages of reduced computation time and the flexibility of expansion against the minor loss in performance when considering this approach.

We developed a lightweight model capable of localizing humans while concurrently segmenting open, traversable areas in warehouses. Depending on the hardware setup, one has the option to switch between larger or smaller backbones, grounded in the YOLOv8 architecture, and can opt to utilize sensors that offer depth data alongside RGB image to further enhance localization accuracy, fully utilizing our proposed model. If desired, the two tasks can also be separated to counteract the slight performance dip, increasing, however, the computational requirements. In essence, our solution presents a versatile perception module for mobile robots, suitable for standalone use or as an integrated supervisory layer for commercial platforms.

### 5.2 Future research directions and potential applications

The development of a specialized human localization algorithm for industrial mobile robots, combined with the ability to perceive free space promises a multitude of potential applications that could transform existing operations and bring enhanced efficiency and safety to the industrial workplace. By accurately predicting the locations of operators, mobile robots can dynamically avoid collision paths significantly minimizing the risk of accidents. In addition, the increased understanding of human location and movement fosters a more harmonious coexistence of humans and robots, which has a significant role in the operator’s mental health.

Using a 2D pose estimation approach to deliver 3D localization prediction compared to 3D bounding box prediction has the advantage of a richer representation of humans, which opens up the potential of extracting even more information including human intention, trajectory prediction, and status of awareness with respect to the robot. We plan on exploring this field in our future works. Although our design principle targeted low-energy consumption platforms, we trained and tested our networks on high-end hardware. With the ongoing advancements in the field, which make more resources available at reduced costs, we anticipate that our proposed solution can be adapted for even more resource-constrained devices, which would enhance scalability. Consequently, we aim to further decrease inference times by exploring network variations and employing optimization strategies specifically for edge devices, including quantization and layer and tensor fusion.

## Data Availability

The datasets presented in this article are not readily available because corporate secrecy confidentiality. Requests to access the datasets should be directed to dtai@novonordisk.com.
